# Ensemble size perception as a case study of the bounds of adaptation

**DOI:** 10.3758/s13423-026-02895-7

**Published:** 2026-03-19

**Authors:** Sam Clarke, Rachel Olugbusi, Sami R. Yousif

**Affiliations:** 1 Department of Philosophy, Mudd Hall, 3709 Trousdale Parkway, Los Angeles, CA 90089 USA; 2 Department of Psychology and Neuroscience, University of North Carolina at Chapel Hill, Chapel Hill, NC USA; 3 Department of Psychology, Ohio State University, Columbus, OH USA

**Keywords:** Visual adaptation, Ensemble perception, Size perception

## Abstract

Repulsive adaptation effects are widely assumed to obtain for all perceptually represented dimensions. However, the ubiquity of adaptation effects within perception remains untested. We examined ensemble size adaptation as a case study to probe whether adaptation occurs for all perceptually encoded properties. Across four experiments, we investigated whether observers adapt to average size and/or cumulative size of dot arrays. In Experiments 1a, 1b, and 1c participants adapted to displays varying in cumulative and/or average dot size, then judged either the average dot size (1a) or the cumulative dot size (1b) of paired test displays. Results revealed robust adaptation to cumulative size but not average size, regardless of task instructions, and even when confounds with brightness were controlled for (1c). Experiment 2 tested “reverse” adaptation to displays containing smaller average and/or cumulative dots size and, again, found adaptation effects for cumulative size only. The observed lack of adaptation to average size across each of these experiments forces a reinterpretation of previous studies that have investigated size adaptation and calls into question arguments which have assumed adaptation to be universal within perception, given a large body of work that finds average size to be perceptually encoded.

## Introduction

The visual system “adapts” to a wide range of features. You have probably experienced at least one form of visual adaptation yourself: If you stare at a bright red square for 20 s, then shift your gaze to a white wall, you will visually experience a green square in the retinotopic location of the original item. The green aftereffect that you experience is an example of *color* adaptation, a repulsive visual aftereffect that occurs following prolonged exposure to its opponent color. You may have also experienced repulsive visual aftereffects to things like motion: If you stare at a waterfall and then avert your gaze to a stationary riverbank, you will often vividly experience motion. In fact, adaptation of this sort is not limited to visual perception. Such aftereffects are common to all perceptual systems (see, e.g., Calzolari et al., 2017; Dalton, 2000; McBurney & Pfafmann, 1963; McBurney et al., 1972).

**Fig. 1:**
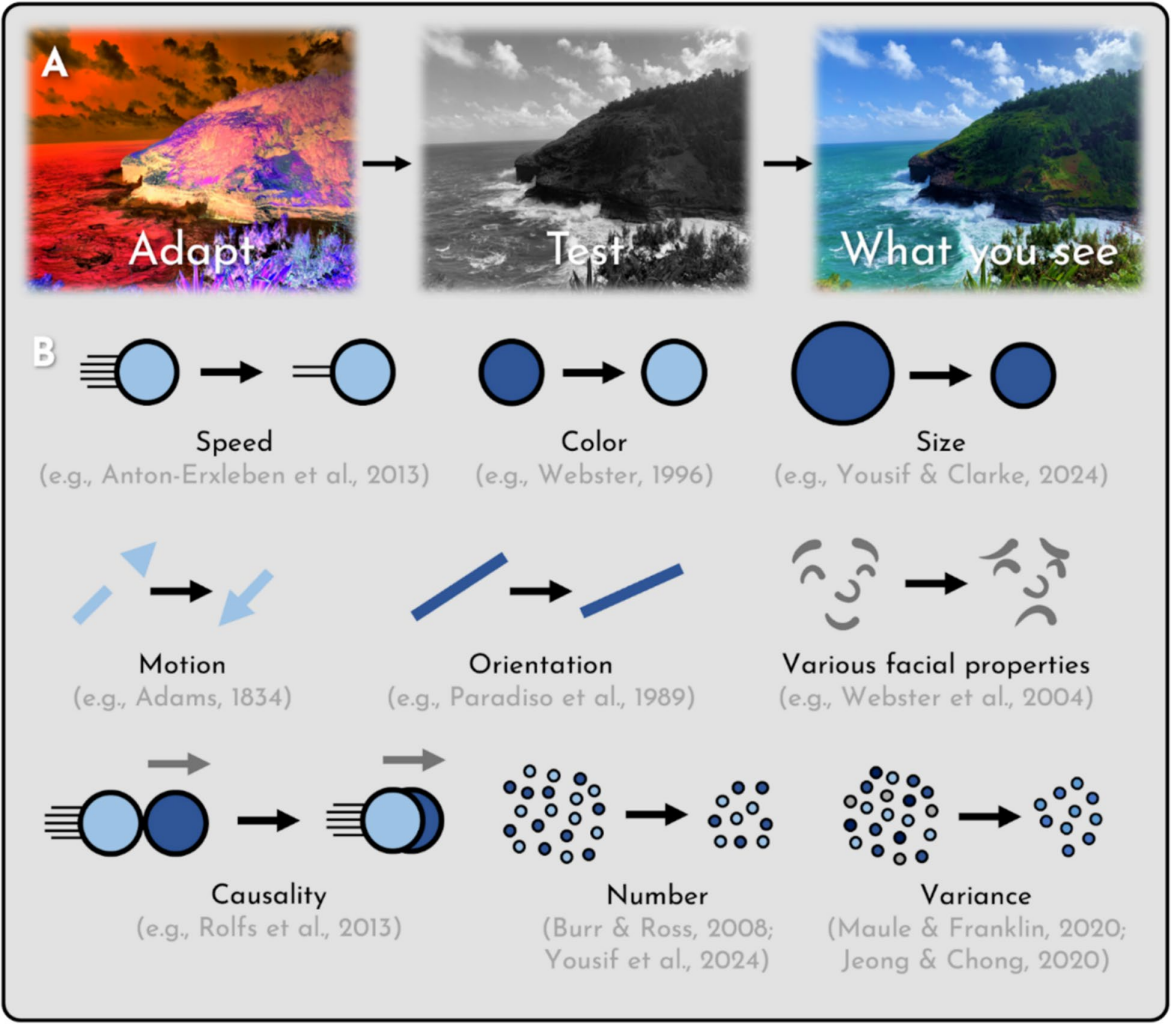
What is adaptation? (**A**) An example of color adaptation, a canonical instance of visual adaptation. (**B**) Illustrations of various other instances of visual adaptation

How pervasive are these adaptation effects? In recent work, considerable attention has been paid to the issue of whether adaptation effects are uniquely perceptual. This is prompted by many prominent researchers assuming as much (cf. Clarke & Yousif 2026; Helton 2016; Phillips & Firestone 2023; Smortchkova 2020). For instance, it has been argued that since number adaptation exists, number must be a “primary visual attribute,” represented in vision, much like color and shape, and not merely encoded at the level of post-perceptual thought (Anobile et al., 2016; cf. Yousif & Clarke, forthcoming). Similar arguments have been made with respect to other, contested visual properties that exhibit adaptation, including causality (Rolfs et al., 2013), gender, emotion, race (Webster et al., 2004), and variance (Maule & Franklin, 2020) (see Fig 1).

In the present treatment, we consider the inverse of this claim. Instead of asking whether adaptation can and does occur outside of perception, we ask: Is adaptation *ubiquitous* within perception, applying to *all* perceptually represented properties? In other words, are there perceptually represented dimensions that fail to adapt?

This question has received less attention than the first. However, a common suggestion seems to be “No,” with researchers routinely assuming that all perceived dimensions adapt. For instance, Block (2014) argues that since we do not adapt to learnt or enculturated properties (but see Clarke & Yousif, 2026), these properties do not feature in the contents of perception and perceptual experience, *pace* salient arguments to the contrary (Siegel 2010). Likewise, Burr et al. (2025) assume as much when defending their claim that humans adapt to number against recent critiques (Yousif et al., 2024, 2025). According to them we should expect that number adaptation is real, for the simple reason that “If [number] did not adapt it would be unique amongst perceptual attributes, worthy of very special attention” (p. 5).

What justifies this assumption that adaptation is ubiquitous in perception, applying to all perceptually represented dimensions? One motivation seems to be the simple observation that readily appreciable and phenomenologically striking adaptation effects obtain for many canonical visual and perceptible properties – including color, orientation, brightness, motion, temperature, and weight. But while this much is consistent with the ubiquity of perceptual adaptation, it fails to establish as much (see, e.g., Phillips & Firestone, 2023). Thus, a question arises: Is adaptation ubiquitous in perceptual representation? Or are there perceptual contents that *fail* to exhibit adaptation?

To answer this question, we take ensemble size adaptation as a case study. One reason for our interest in size adaptation is that, while size exhibits perceptual adaptation (see, e.g., Kreutzer et al., 2015; Pooresmaeili et al., 2013; Yousif & Clarke, 2024; Zeng et al., 2017), it is not yet clear what *kind(s)* of size adaptation occur. Most prior studies on the phenomenon focus entirely on adaptation to the size of individual objects, though one prior study has reported finding that people adapt to *average* size (i.e., of a collection of dots) as well (Corbett et al., 2012). Given the received view that adaptation is pervasive in perception, it is natural to expect that adaptation would obtain for ensemble properties like average size, since many ensemble properties are taken to be perceptually represented (for review, see Whitney & Yamanashi Leib, 2018). This is particularly true of average size, which has been studied in this context extensively (Albrecht & Scholl, 2010; Ariely, 2001; Chong & Treisman, 2003, 2005; Marchant et al., 2013; Raidvee et al., 2020; Sweeney et al., 2015), and is widely regarded as a feature that is represented by perceptual systems. To wit: Corbett and colleagues (2012) open their paper by stating, simply: “The visual system rapidly represents the mean size of sets of objects” (p. 211). Albrecht and Scholl (2010) open their work in a similar fashion: “Beyond processing individual features and objects, the visual system can also efficiently summarize scenes – for example, allowing observers to perceive the average size of a group of objects” (p. 560). The rationale for viewing average size as a perceptual dimension, we take it, is that average size is processed *quickly* (i.e., with sub-second exposures to a set), *automatically* (e.g., without explicit direction), and in a relatively *encapsulated* manner (i.e., independently of one’s knowledge of the situation) – key signatures of visual processing (see Hafri & Firestone, 2021; Scholl & Gao, 2013).

Prior work on ensemble adaptation has, however, failed to tease apart adaptation to average size from adaptation to cumulative size (Corbett et al., 2012). Given the available evidence, it may then be that people adapt to average size, or cumulative size, or both. This distinction matters if we are interested in understanding the nature and scope of perceptual adaptation. Suppose, for instance, that people adapt to cumulative size but not average size. Would that mean, as per the logic employed by those hypothesizing that adaptation is diagnostic of perception (see Block, 2023; Burr et al., 2025), that cumulative size but not average size is perceived? And if that is the case, what should be made of the typical and widely supported assertion that ensemble features like average size are perceptual attributes?

### Current study: The bounds of adaptation

The present study asks whether ensemble representations of average size and/or cumulative size exhibit perceptual adaptation.

We can imagine three possible outcomes from our experiments. One outcome is that people adapt to both average and cumulative size. Such a pattern may support the view that any visual property is or can be prone to adaptation, and that both average and cumulative size feature in the contents of human vision. Another possible outcome is that people adapt to average size but not cumulative size. Such a pattern of results might be expected insofar as average size has been studied extensively as a perceptual property, while cumulative size has not. However, a final possibility is that people adapt to cumulative size but not average size. This pattern might be the most surprising, since prior work has assumed that average size is perceived (and thus should, given claims of perceptual adaptation’s ubiquity, exhibit adaptation) where less attention has been paid the perceptual encodability of cumulative size.

## Experiments 1a, 1b, and 1c: Cumulative versus average size

**Fig. 2:**
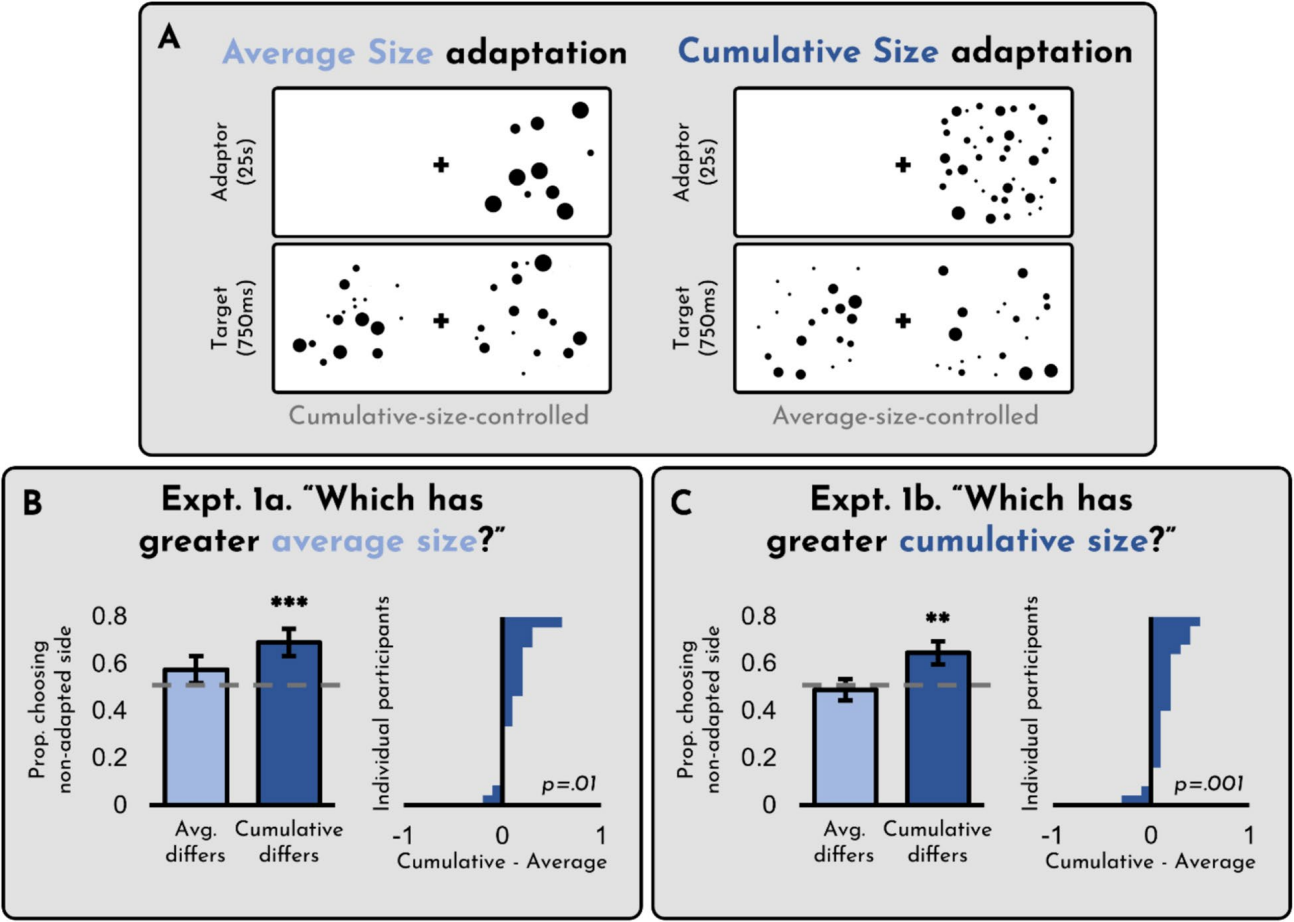
(**A**) Design of Experiments 1a and 1b. Both experiments included the same trial types; they differed only in what participants were asked to evaluate. (**B**) Results of Experiment 1a. (**C**) Results of Experiment 1b. For both (**B**) and (**C**), the x-axis on the right panel reflects the difference between the two conditions on the left, such that any bar to the right indicates a participant who showed a greater cumulative size adaptation effect than an average size adaptation effect. Error bars represent +/- 1 SE

**Fig. 3:**
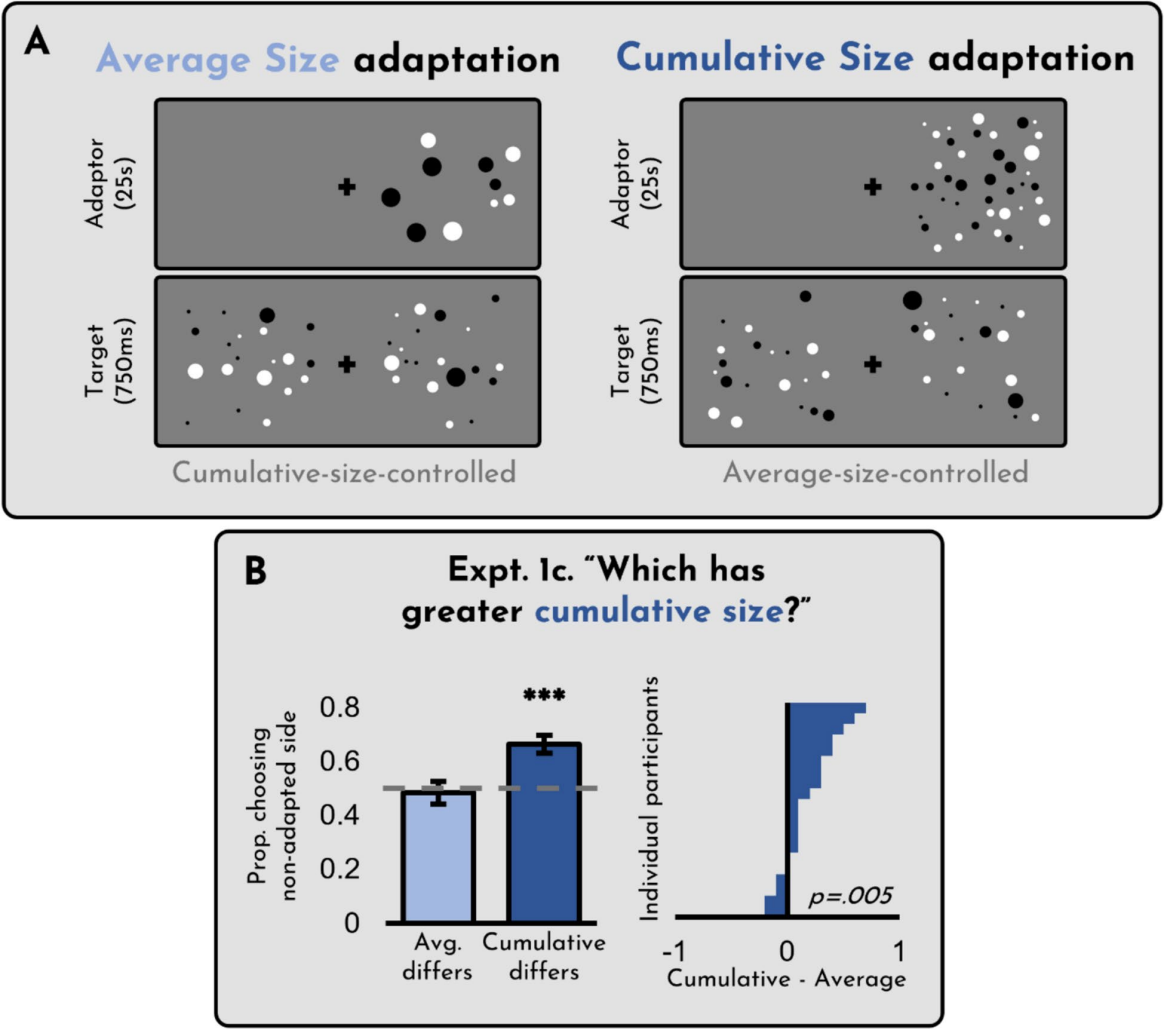
(**A**) Design of Experiment 1c. (**B**) Results of Experiment 1c. The x-axis on the right panel reflects the difference between the two conditions on the left, such that any bar to the right indicates a participant who showed a greater cumulative size adaptation effect than an average size adaptation effect. Error bars represent +/- 1 SE

To evaluate ensemble size adaptation, we had participants adapt to displays that varied in cumulative size and average size (see Fig. 2A). We tested whether they adapted to one or both of these dimensions. Critically, we ran two different versions of the experiment: In Experiment 1a, participants were asked to evaluate average size, while in Experiment 1b, participants were asked to evaluate cumulative size. In both cases, they saw the same displays; all that changed was the instructions. This design allowed us to independently assess (a) whether people adapt to cumulative versus average size, and also (b) whether this adaptation depends on the explicit instructions they are given. After running both versions of these experiments, an anonymous reviewer questioned whether the observed effects could be explained by either brightness or contrasty energy. To address this concern, we subsequently added a third version of the experiment, identical to Experiment 1b except that the displays consisted of intermixed black and white dots – a control which is standardly seen to control for these confounds (see Fig. 3A).

### Methods

The design, sample size, and analysis plan were all pre-registered in advance. The pre-registration for this experiment as well as raw data for both experiments can be found on our Open Science Framework (OSF) page: https://osf.io/k2qm6/overview?view_only=015510b4449d47b9b847c7509e0b4c66.

#### Participants

Sixty undergraduate students (20 per experiment) participated through a volunteer participant pool in exchange for course credit or monetary compensation. All participants were adults, aged 18 years or older, who were proficient speakers of English. There were no exclusions. The study was approved by the relevant Institutional Review Board (IRB).

#### Stimuli

For Experiments 1a and 1b, stimuli were black dot arrays presented on a white background, positioned to the left and right of a central fixation cross. For both cumulative and average size, we manipulated cumulative/average diameter rather than true size. This is because a wide range of work has demonstrated that perceived size is roughly equivalent to diameter size rather than true area (see, e.g., Anderson & Cuneo, 1978; Carbon, 2016; Ekman & Junge, 1961; Yousif et al., 2020; Yousif & Keil, 2019, 2021; cf., Teghtsoonian, 1965). This choice is also consistent with prior work on size adaptation (Corbett et al., 2012). All subsequent units mentioned reflect pixels of diameter length. For Experiment 1c, all the stimulus parameters were the same except that half of the dots were black and half were white (randomly chosen). The background was gray. See Fig. 3A.

The parameters of the adaptors and targets were designed to dissociate effects of cumulative size and average size within a single stimulus set. To simplify the experiment, all target stimuli had exactly 20 dots. Most target stimuli had a total size of 400 units. Forty percent of the time, one of the target stimuli had a total size of 480 units (equally often on the left and right side), such that there were functionally five possible true area combinations: [480, 400], [400, 400], [400, 400], [400, 400], [400, 480]. (Three of these five combinations were equal; this was because we wanted the equal trials to be overrepresented.) This allowed us to assess whether participants were properly sensitive to true differences in size. Individual dots could be as small as 10 pixels in diameter or as large as 50 pixels in diameter. Locations of dots were randomized with the constraint that they could not appear within 20 pixels of another dot (from edge to edge). All stimuli, exactly as they appeared for participants, have been made available on our OSF page.

There were four possible configurations for the adaptor stimuli. Relative to the corresponding target stimulus, it could have had:

- The same total area, but a higher average dot area,
- A higher total area, but the same average dot area,
- A higher total area and a higher average dot area, or
- The same total area and the same average dot area.

The adaptor appeared on the left and right sides equally often. Therefore, there were: four possible adaptor configurations, two possible adaptor sides (left, right), and five possible target area combinations. In practice, this meant that the number of dots in the adaptor stimuli could be either 10, 20, or 40. This setup resulted in a total of 40 trials. The order of trials was fully randomized for each participant. Participants completed a single representative practice trial before starting the main task.

#### Procedure and design

Prior to beginning the task, participants were given basic instructions. They were explicitly told whether they were to judge average versus cumulative size (depending on which condition they were assigned to; see below). They were shown some example displays as well as one example of a full adaptation trial (the data from which were not recorded). Throughout the instructions, participants were able to ask clarification questions as needed. They began the task once they indicated that they fully understood what they were meant to do. All participants viewed the same trials in a unique random order, each beginning with a 25-s adaptation phase, followed by a 750-ms test display, after which the screen would remain blank until a participant indicated a response. They were instructed to press the “Q” key if they thought the left side was greater in average/cumulative size, and to press the “P” key if they thought the right side was greater in average/cumulative size.

Participants were randomly assigned to one of two conditions: In Experiment 1a, they were asked to indicate which side had the greater *average* dot size; in Experiments 1b and 1c, they were asked to indicate which side had the greater *cumulative* dot size. Note that in all experiments, however, they experienced trials in which the adaptor varied with respect to both cumulative and average size. Stimulus presentation, randomization, and the debriefing protocol were identical across all three groups. The only difference between 1a and 1b was whether they were told to evaluate cumulative or average size, and the only difference between 1b and 1c was the color of the stimuli.

### Results and discussion

The results of Experiments 1a and 1b can be seen in Fig. 2. First, we checked whether participants successfully identified the display with greater cumulative/average size for the trials in which there was a correct answer. They did. In Experiment 1a, overall accuracy was 74% (SD = 24%; *t*(19) = 4.47, *p* < 0.001, *d* = 1.00), and in Experiment 1b, overall accuracy was 78% (SD = 12%; *t*(19) = 10.72, *p* < 0.001, *d* = 2.40). Thus, we can be confident participants were completing the task correctly.

Of the 40 trials in each experiment, ten of them represent a pure test of cumulative size adaptation, and another ten represent a pure test of average size adaptation. The results reported below are for the critical trials in each case. As is evident from the figure, when participants were asked to assess *average* size (Experiment 1a), we found an adaptive effect of cumulative size (*t*(19) = 4.34, *p* < 0.001, *d* = 0.97), but not average size (*t*(19) = 1.60, *p* = 0.13, *d* = 0.36), (difference: *t*(19) = 2.82, *p* = 0.01, *d* = 0.63). The same was true when participants were asked to assess *cumulative* size (Experiment 1b): we found a clear adaptation effect on cumulative size (*t*(19) = 2.80, *p* =.012, *d* = 0.63), but not average size (*t*(19) = 0.43, *p* = 0.67, *d* = 0.10; difference: *t*(19) = 3.74, *p* = 0.001, *d* = 0.84). In other words, regardless of the dimension participants were asked to evaluate, they only exhibited a repulsive aftereffect (i.e., were more likely to choose the opposite side of the display) when the adaptor had a larger cumulative size than the corresponding target. In all of these cases, the results are qualitatively identical for all subsets of the data, whether, for instance, we analyze the trials in which there was a true difference in size or not.

We think these results are unlikely to be explained by confounds with features like number, for several reasons. First, we believe that number adaptation is unlikely to be genuine (Yousif et al., 2024, 2025). Second, it is not clear why adaptation to number would influence judgments of size, since proponents of number adaptation are committed to the view that while observers experience a reduced sense of number following number adaptation, “no particular dots seem to be missing” (Burr & Ross, 2008; p. 426; but see Cicchini et al., 2016, and Yousif & Keil, 2020, for the possibility that this occurs via a congruency effect). Finally, and most importantly, we believe that the data contradict this concern: On trials where the adaptor has a greater average value *and* a greater cumulative value (but no difference in number), there is still a robust adaptation effect (Experiment 1a: *t*(19) = 3.38, *p* = 0.003, *d* = 0.76; Experiment 1b: *t*(19) = 4.49, *p* < 0.001, *d* = 1.00). Thus, the most parsimonious interpretation of these data is that people adapt to cumulative size, but not average size, regardless of number.

The results of Experiment 1c can be seen in Fig. 3B. As is evident from the figure, we again observed a robust cumulative size adaptation effect (*t*(19) = 4.82, *p* < 0.001, *d* = 1.08) but no average size adaptation effect (*t*(19) =.36, *p* = 0.72, *d* =.08). These results indicate that the cumulative size adaptation effect is not caused by a confound with brightness or contrast energy. Insofar as the black-and-white stimuli are in keeping with the ‘state of the art’ in stimulus design for size and number adaptation studies (see, e.g., Burr & Ross, 2008; Pooresmaeili et al., 2013; Yousif et al., 2024), there seems to be considerable evidence in favor of cumulative size adaptation driving our observed results.

## Experiment 2: “Reverse” adaptation

In Experiments 1a and 1b, we found that adaptation to cumulative size produced a repulsive effect: participants were more likely to judge the collection on the side opposite to that of the adaptor to be larger. However, in the critical trials from those experiments the adaptor was always larger in terms of its average/cumulative size than the target stimuli. In Experiment 2, we tested whether “reverse” adaptation, adaptation to a smaller array, would produce a comparable repulsive (this time *upward*) aftereffect.

### Methods

This experiment was identical to Experiments 1a, 1b, and 1c except as noted below.

The parameters of the adaptors and targets were designed to dissociate effects of cumulative size and average size within a single stimulus set, as in Experiments 1a and 1b. To simplify the experiment, all target stimuli had either 20 or 40 dots. Most target stimuli had a total size of 800 units. Forty percent of the time, one of the target stimuli had a total size of 960 units (equally often on the left and right side), such that there were five different target area combinations: [960, 800], [800, 800], [800, 800], [800, 800], [800, 960]. This allowed us to assess whether participants were properly sensitive to true differences in size. Individual dots could be as small as 10 pixels in diameter and as large as 50 pixels in diameter. Locations of dots were randomized with the constraint that they could not appear within 20 pixels of another dot (from edge to edge). All stimuli, exactly as they appeared for participants, have been made available on our OSF page.

As in Experiments 1a, 1b, and 1c, there were four possible configurations for adaptor stimuli. Relative to the corresponding target stimulus, it could have:

- The same total area, but a higher average area;
- A higher total area, but the same average area;
- A higher total area and a higher average area; or
- The same total area and the same average area.

The adaptor appeared on the left and right sides equally often. Therefore, there were: four possible adaptor configurations, two possible adaptor sides (left, right), and five possible target area combinations. In practice, this meant that the number of dots in the adaptor stimuli could be either 10, 20, or 40. This setup resulted in a total of 40 trials. The order of trials was fully randomized for each participant. Participants completed a single representative practice trial before starting the main task.

In this experiment, all adaptors were smaller than the target arrays, allowing us to test whether adaptation to smaller cumulative sizes would produce comparable aftereffects.

### Results and discussion

**Fig. 4:**
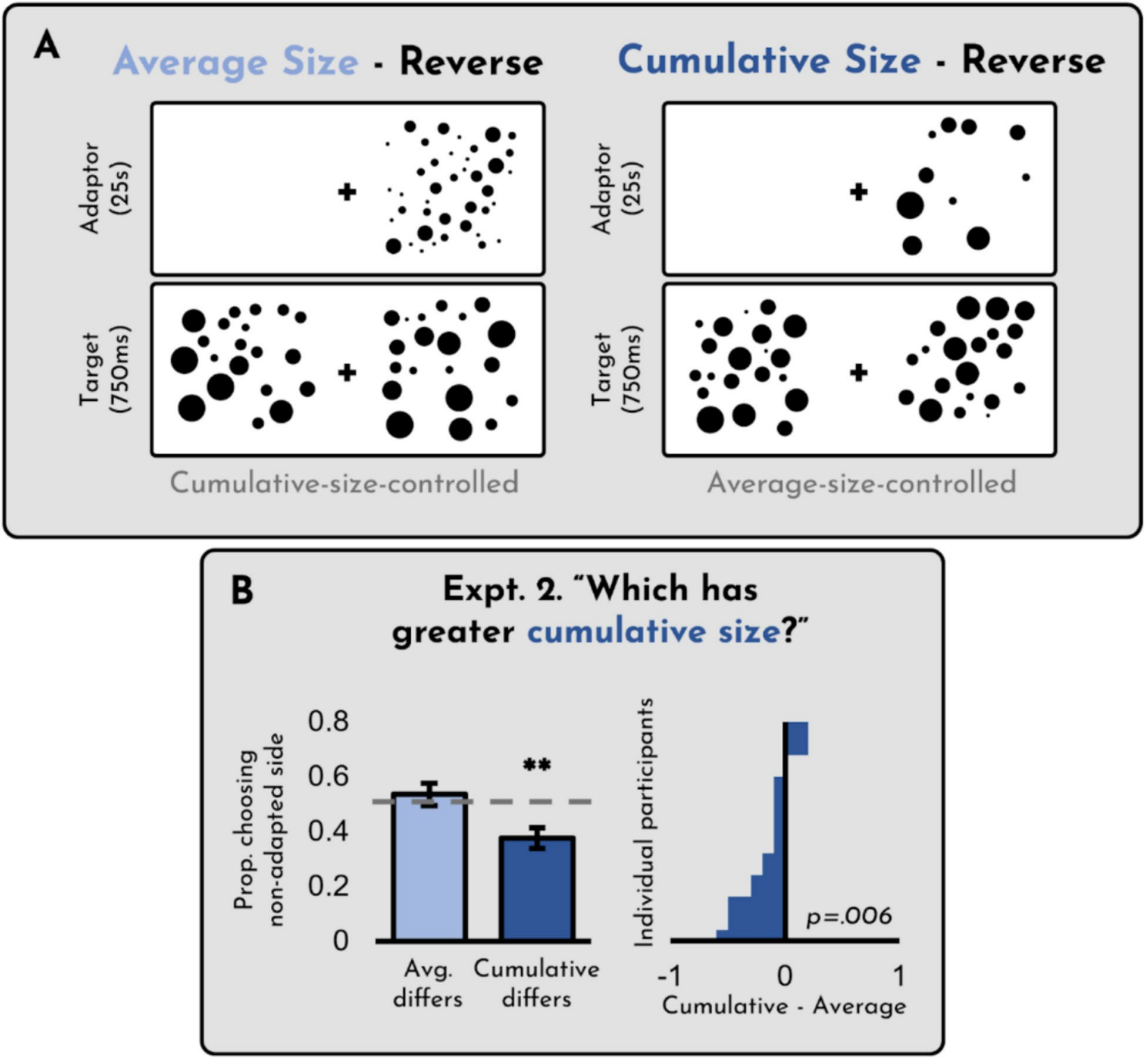
(**A**) Design of Experiment 2. (**B**) Results of Experiment 2. The x-axis on the right panel reflects the difference between the two conditions on the left, such that any bar to the right indicates a participant who showed a greater cumulative size adaptation effect than an average size adaptation effect. Error bars represent +/- 1 SE

The results of Experiment 2 can be seen in Fig. 4. First, we checked whether participants successfully identified the display with more cumulative/average size for the trials in which there was a correct answer. Overall accuracy was 90% (*SD* = 9%; *t*(19) = 21.15 *p* < 0.001, *d* = 4.73).

As is evident from the figure, we found a “reverse” adaptation effect for cumulative size (cumulative: *t*(19) = 3.32, *p* = 0.004, *d* = 0.74) but not average size (*t*(19) =.85, *p* = 0.41,* d* = 0.19; difference: *t*(19) = 3.05, *p* = 0.007, *d* = 0.68). That is, when the adaptor had a *lower* cumulative size than the corresponding target, participants were more likely to indicate that the adapted side was *greater* in cumulative size. However, when the adaptor had a *lower* average size than the corresponding target, participants were no more likely to indicate that the adapted side was *greater* in average size.

The existence of reverse cumulative size adaptation is surprising, given that prior work has reported effects of average size adaptation, but not cumulative size adaptation (Corbett et al., 2012). It is also intriguing given that many other cases of high-level visual adaptation exhibit only unidirectional or asymmetric effects (see Pooresmaeli et al., 2013; Yousif et al., 2024, 2025). We therefore find these results particularly compelling, as this form of adaptation appears to be more robust than comparable cases of adaptation.

## General discussion

Across four experiments, we have shown that (1) when observers were asked to evaluate *average* size, they readily adapt to cumulative but not average size (Experiment 1a); (2) when observers were asked to evaluate *cumulative* size, they also readily adapt to cumulative but not average size (Experiment 1b); (3) the effect of cumulative size survives controls that carefully account for stimulus features like contrast and brightness (Experiment 1c); and (4) observers also exhibit “reverse” cumulative (but not average) size adaptation, such that adapting to a display of a lower cumulative size (but middling average size) exerts upward adaptive pressure on a subsequent stimulus of higher cumulative size (Experiment 2). Collectively, these results suggest that people robustly adapt to some, but not all, properties of perceived ensembles. Furthermore, they conflict with prior work on average size adaptation (Corbett et al., 2012), and perhaps most surprisingly, indicate that the existence of this adaptation is relatively impervious to the instructions that participants are given.

These results, thereby, point to several distinctive and surprising signature limitations on size adaptation. For us, though, size adaptation is really just a “case study”: What we really care about is *ensemble adaptation* more broadly – adaptation that occurs not over the visual properties of an individual item but over the properties of a *set* of items (e.g., the cumulative size of a collection of dots, or their average hue, or their number). Prior work has investigated instances of ensemble adaptation including adaptation to number (Burr & Ross, 2008), texture density (Durgin, 1995), average motion direction (Kar & Krekelberg, 2014), average size (Corbett et al., 2012), variance (Maule & Franklin, 2020), and even cumulative value (Clarke & Yousif, 2026), yet most of this work fails to engage with the theoretical significance of such adaptation (but see Corbett et al., 2012).

The significance is not lost on Bayne and McLelland (2019), who note that adaptation may help to adjudicate whether ensemble perception should in fact be considered a genuine case of “perception.” Citing work from Block (2014; see also Block, 2023) and other philosophers (Fish, 2013), they note that “...the strongest evidence for a perceptual view of ensemble coding is adaptation” (p. 738). On such a view, evidence of adaptation to cumulative size may be viewed as tantamount to proving that cumulative size is represented by the visual system directly. Indeed, this exact argument is made about cases like number adaptation (Burr & Ross, 2008).

That said, Bayne and McLelland go on to clarify that they are skeptical of the link between adaptation and perceptual processing: “...our own view is that it is very much an open question whether adaptation is a mark of perception” (p. 738). We agree (see Clarke & Yousif, 2026; Yousif et al., 2024, 2025; Yousif & Clarke, 2024; Yousif & Clarke, forthcoming; see also Phillips & Firestone, 2023). Nevertheless, we think it is clear that adaptation has a role to play in understanding perceptual (and plausibly perceptual) processes.

In the same way that adaptation may have a role to play in helping us to answer important theoretical questions about ensemble representation, ensemble representation may help us to answer important theoretical questions about adaptation. Namely: What *is* adaptation (see Clarke & Yousif, 2026; Yousif & Clarke, forthcoming)? Are there reasons to expect that certain features should exhibit adaptation but not others? Simple as these questions may seem (if we think that ensemble representations are perceptual, and perceptual attributes exhibit adaptation, then of course ensemble representations would exhibit adaptation!), the present results are just one example of how this picture is more complex than many assume.

On the adaptation side of the coin, these results demonstrate a clear case in which participants consistently fail to adapt to a widely accepted perceptible feature (average size; see Albrecht & Scholl, 2010; Ariely, 2001; Chong & Treisman, 2003, 2005; Marchant et al., 2013; Raidvee et al., 2020; Sweeney et al., 2015). The failure to observe adaptation in this case is noteworthy in a landscape where it is routinely assumed that every perceptual feature under the sun exhibits adaptation (and, perhaps, even features that are not plausibly perceived; see Clarke & Yousif, 2026). Indeed, the fragility of average size adaptation may indicate that it is likely to be a cognitive rather than perceptual effect, if indeed it can be found to obtain.

One might be tempted to dismiss our single null effect as an anomaly, given robust evidence of ensemble adaptation in other cases (see Burr & Ross, 2008; Corbett et al., 2012; Durgin, 1995; Maule & Franklin, 2020). However, we think that each such case must be examined carefully. We have recently argued, for instance, that there are many unanswered questions regarding both size adaptation (Yousif & Clarke, 2024) and number adaptation (Yousif et al., 2024, 2025). Despite seemingly overwhelming evidence in favor of number adaptation, we argue that this evidence either (a) can be explained by other visual mechanisms that all parties must accept as actual, or (b) is insufficiently robust to license the strong claims that have been made about it.

### Truly “visual” adaptation?

As we (see Clarke & Yousif, 2026; Yousif et al., 2024, 2025; Yousif & Clarke, 2024) and others (Bayne & McLelland, 2019; Helton 2016; Phillips & Firestone, 2023; Smortchkova 2020) have pointed out, there is reason to be skeptical about any strong link between adaptation and perception. Traditionally, adaptation is considered to be diagnostic of perceptual processing insofar as (a) the resulting effects are phenomenologically compelling, such that any observer can (literally) see them with their own eyes, and (b) they are retinotopic (i.e., specific to a location on the retina; see Kominsky & Scholl, 2020; Rolfs et al., 2013) or at least spatiotopic (i.e., specific to a location in external coordinates; see Arrighi et al., 2014; Block, 2023; Clarke & Yousif, 2026). In this case, it is not obvious that the effects documented here are phenomenologically compelling (although, this proves to be common for higher-level adaptation effects, which rarely work well as demonstrations when low-level confounds are controlled for). And while these effects are spatially selective to some degree, we did not go so far as to test whether these effects are retinotopic. Thus, a question remains not only about what kinds of ensemble adaptation are genuine, but also whether such adaptation is indeed indicative of perceptual processing. The fragility of average size adaptation effects (if they exist at all) relative to cumulative size adaptation effects may indicate that the former is more likely to result from cognitive processes rather than perceptual ones.

## Limitations and future directions

Here, we have dissociated cumulative size from average size. However, fully separating quantity dimensions is notoriously tricky. Some even consider this problem intractable (see Leibovich et al., 2017); but see DeWind et al., (2015). One might think that by dissociating average size and cumulative size, we necessarily created a confound with number, or density, or convex hull – or even more pernicious confounds like brightness or contrast. Yet there are a few reasons that we stand by our conclusions. First, the most similar experiments to our own are those of Corbett and colleagues (2012). Those experiments would also be subject to the same concerns. If anything, our experiments move beyond the current “state of the art” in this domain. This is particularly true of Experiment 1c, which uses alternating black/white dots to equate for overall brightness and contrast. Second, even if it was the case that some third variable explained the *presence* of cumulative area adaptation – a suggestion we accept is possible in principle, even if unlikely in practice – this would not explain the observed *absence* of average size adaptation. Our theoretical conclusions lean much more heavily on the latter finding. We think these other variables are worth taking seriously (and we have argued as much in related work; see Yousif et al., 2024; Yousif & Clarke, 2024), but we do not think they prevent us from comparing two related dimensions.

In short: We know of no case of high-level adaptation that can be definitively dissociated from all its low-level constituents, but we believe that our experiments offer a fair, clean dissociation between average size and cumulative area. Even if one doubts whether we have demonstrated proof of cumulative size adaptation, they ought to see that our results do meaningfully reshape our understanding of ensemble size adaptation (via direct comparison to Corbett et al., 2012), and place the onus on those who routinely assert that adaptation is ubiquitous in perception.

How seriously should we take the absence of average size adaptation? As we have framed it here, we find the absence of average size adaptation to be telling, since (a) we take it that most people consider average size to be a dimension that is represented by the visual system, and (b) we find no hint of such adaptation once we control for cumulative size. But, of course, there is always the possibility that average size adaptation may exist in other contexts. Consider the following from Webster (2016):

“Can we adapt to anything? Despite the rich varieties of visual aftereffects, there are also limits to how the visual system can adapt. One obvious limit is set by the selectivity of the adaptation. If different patterns produce the same net activity within the sites controlling the adaptation, then they will not induce different states of adaptation, even if the stimuli are distinguishable” (p. 550).

We are not sure what it would mean in practice to say that certain patterns “produce the same net activity,” but we do accept in principle the possibility that, insofar as the neural mechanisms of adaptation are poorly understood, there are plenty of ways that adaptation might fail to manifest in certain contexts even when it is unambiguous in others. For this reason and others, we do not think our results could possibly be taken as definitive evidence that average size adaptation does not exist. Instead, we think our results should be viewed as a strong indication that we should approach average size adaptation with skepticism. These uncertainties could be addressed in future work.

## Conclusion

Ensemble representation and adaptation are both key theoretical constructs in modern vision science. The study of one constrains the other: Insofar as ensemble properties are regarded as perceptual, as they often are, and insofar as adaptation is expected to occur over all perceptual properties (see, e.g., Burr et al., 2025), the presence or absence of adaptation to any putative ensemble property bears on debates about ensemble representation, adaptation, or both. Whether cumulative size adaptation is truly visual in nature remains an open question (Clarke & Yousif 2026). Nevertheless, our results indicate that properties that are typically seen to be represented in perception (average size) can fail to adapt. Findings like these bring us one step closer to a complete understanding of adaptation – and blunt the force of recent arguments premised on the assumed ubiquity of adaptation effects within perception.

## Data Availability

https://osf.io/k2qm6/overview?view_only=015510b4449d47b9b847c7509e0b4c66.
